# Successful perioperative preparation of a child with autism spectrum disorder in collaboration with his school for special needs education: a case report

**DOI:** 10.3389/fpsyt.2023.1162833

**Published:** 2024-01-05

**Authors:** Yuto Arai, Tohru Okanishi, Yuko Nakamura, Yoshihiro Maegaki

**Affiliations:** Division of Child Neurology, Department of Brain and Neurosciences, Faculty of Medicine, Tottori University, Yonago, Japan

**Keywords:** autism spectrum disorder, unique behavior, perioperative management, parents, school for special need education, school information, school cooperation

## Abstract

**Introduction:**

The incidence of autism spectrum disorder (ASD) in children is greater than 1%. Children with ASD show an increased rate of hospital contact for several reasons. Perioperative preparation for children with ASD can be challenging; therefore, obtaining information from patients’ families prior to surgery is important. However, no previous reports have described the collection of information from educational facilities.

**Case report:**

A 12 years-old male patient with ASD was referred for surgery for traumatic dislocation of the left knee joint. Before admission, we obtained valuable information from his parents regarding expected behavioral problems and coping strategies during hospitalization and from his teachers at his school for special needs education. In particular, the information obtained from teachers was specific and practical. Consequently, we could effectively conduct perioperative management based on his specific autistic characteristics.

**Conclusion:**

We report a pediatric case of ASD in which favorable perioperative management was successfully achieved by collecting information before admission from family members as well as teachers at the patient’s school for special needs education. This management may help in future hospital admissions for children with autism.

## Introduction

The estimated prevalence of autism spectrum disorders (ASD) in children and adolescents is approximately 1.5% (1). Children with ASD are more likely to experience medical complexities than their typically developing peers and more frequently utilize the healthcare system (2). Children with ASD also have an increased rate of hospital contact for multiple reasons (3) and an increased risk of adverse events during hospitalization or procedures (4).

Perioperative preparation for children with ASD can be challenging because of their unique individual needs and behavioral differences (5). However, data on perioperative preparation for children with ASD are limited (6), and little is known about the optimal management approaches for patients who require surgical intervention (7). Previous studies have shown that healthcare providers should obtain the necessary information for preparation from the patient’s family members prior to the child’s arrival at the hospital (4, 5). On the other hand, the individual coping methods employed for children with ASD at schools for special needs education, which are employed by non-family members, may be easier to apply for medical staff, who are also not family members and are likely to be more practical for the perioperative preparation of children with ASD. However, there have been no reports on the application of coping methods used in education for the preparation of children with ASD.

Herein, we report a case of pediatric ASD in which favorable perioperative management was successfully achieved by collecting information before admission from family members as well as the teachers at the patient’s school for special needs education.

## Case report

A male patient was referred for surgery for a traumatic dislocation of the left knee joint. The patient was 12 years old with a history of Pitt-Hopkins syndrome (identification of *TCF4* gene mutation). He had underlying ASD and severe intellectual disability (Intelligence Quotient: 31). The patient was treated with aripiprazole (2 mg/day) and as-needed risperidone for irritability associated with ASD. His family consisted of his father, mother, and a 14 years-old brother. He was in the sixth grade at a school for special needs education and had been in a welfare facility for disabled children since the first grade of elementary school. He spent weekdays in the welfare facility and weekends at home. At the age of seven, he fell from a height and was diagnosed with traumatic dislocation of the left knee joint, which was treated conservatively with a brace, considering the difficulty in perioperative management. However, his gait gradually deteriorated, and he experienced frequent falls. Therefore, his family strongly desired surgical intervention, and he was referred to our hospital.

**Figure 1:**
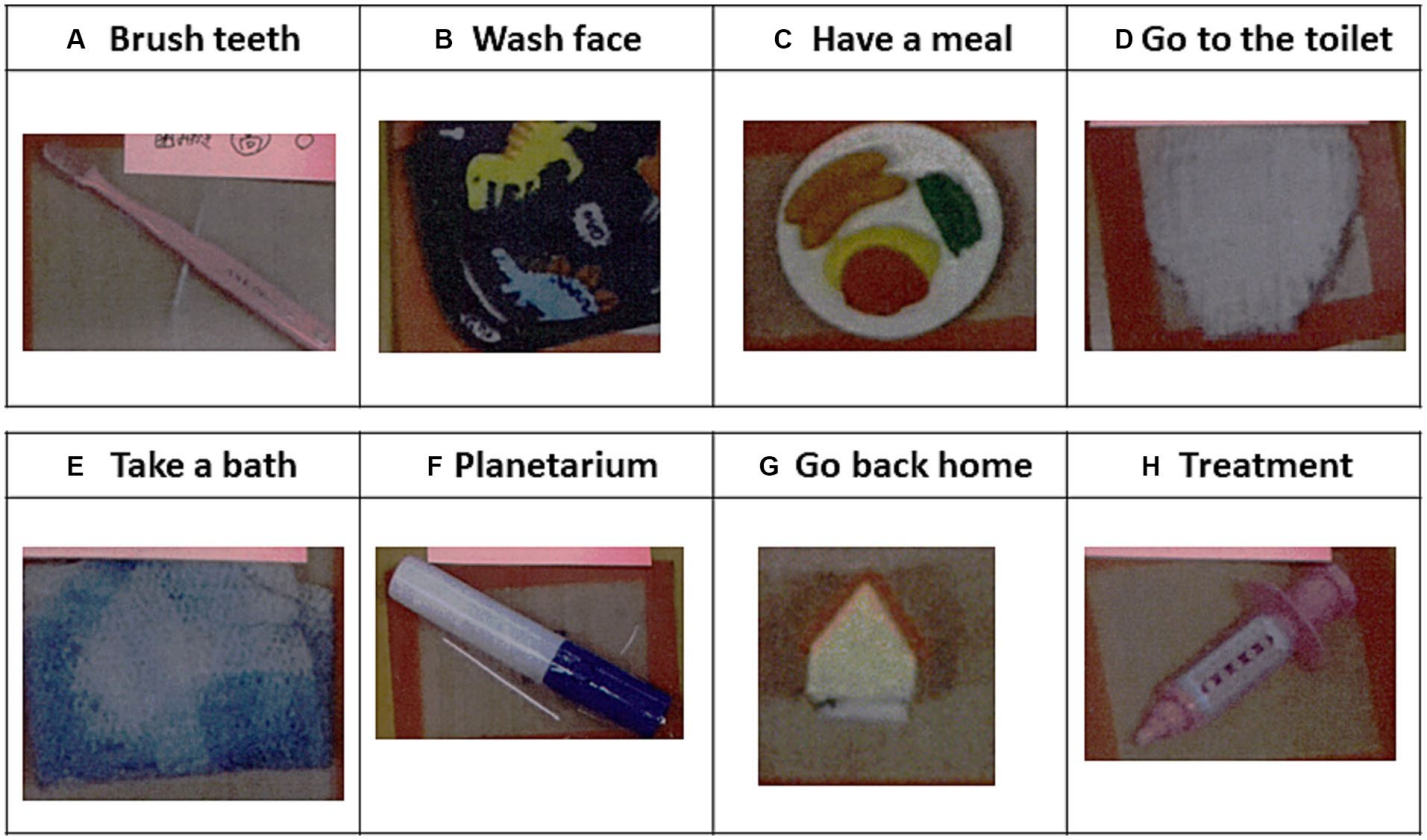
Examples of picture cards used in the school for special needs education. The cards were obtained before admission from the patient’s teachers. Picture cards of **(A)** a toothbrush for brushing teeth, **(B)** a towel for washing the face, **(C)** a cooking plate for eating food, **(D)** a diaper for going to the toilet, **(E)** a pajama for taking a bath, **(F)** a glowing pen for playing with a planetarium, **(G)** a toy house for going home, and **(H)** a toy injection for indicating upcoming medical procedures.

Before admission, we obtained information about the children’s autistic symptoms from his parents and teachers. We obtained information directly from his parents as an outpatient first, and then obtained information from his teachers through online meetings and patient referral documents. The collected information was summarized according to a previously reported protocol (4) (Table 1). In addition, his teachers explained about the picture cards that they typically used (Figure 1). Based on this information, we made the following decisions: (i) the patient was to be admitted in principle with his mother, (ii) when his mother had to go out for some reason, the medical staff would take care of the patient based on the information gathered before hospitalization as summarized in Table 1.

**Table 1:** Summary of information from parents and schools and preparation at the hospital.

Type of behavior	Inputs from family	Inputs from school	Preparations at the hospital
Persistent deficits in social communication	• He does no harm to other people	• He requests by crane phenomenon • He clap his hand twice when he wants to ask • When he refuses, sits still	• Sharing with the medical staff how he makes his wishes known
Restrictive and repetitive interests, behaviors, and activities	• He calms down with a music box and planetarium in the darkroom	• He likes to repeatedly turn cup containers and lids • When putting on clothes, spread them out in the order in which he will be worn • Say “Goshigoshi” when washing his face • When washing the body, indicate body parts to wash every 10 s • Prepare bread by cutting it into sticks	• Used cup containers and lids to comfort • Tried not to break his routine • No whole-body restraint band was used (only fixation of affected lower limb) • Selected a private room that could be darkened with curtains • Used appropriate voice while providing instructions for brushing and washing
Associated mental health problems	• He rocks back and forth when he is in a good mood • When he gets angry, he yells and bites his own hand		• Trying to understand his emotions at an early stage
Sensory hypersensitivity	• He′ll want to pull IV line out • If the room is too small, he will run away	• He hates when we try to clean his eyes • He hates wearing a mask	• Removed IV line as soon as possible • Tried to avoid things that he hated
Intellectual impairment	• He can understand “stand up” and “sit down,” but not “do not move”	• Minimizing words and guiding with card instructions is effective	• Used simple clear language • Used the picture cards given by his school as needed
Epilepsy	• He experiences breath-holding seizures when he gets excited		• Used the music box and planetarium to calm him down • Placed his room near the nurse station to account for the possibility of seizures
IV: intravenous line			

After admission, surgery was performed the following day as scheduled. For analgesic management, continuous intravenous fentanyl injection was administered until the second postoperative day, after which the line was removed and followed by regular oral administration of acetaminophen and diclofenac suppository insertion as needed. Under this analgesic management protocol, the patient was kept calm except on the first day when he cried for pain. He was also emotionally stable with only environmental adjustments based on prehospital information, and he never needed risperidone. The postoperative course was uneventful, and the patient was transferred to a rehabilitation hospital as scheduled. We explained our management methods to the hospital.

## Discussion

We performed perioperative preparation for the treatment of traumatic dislocation of the knee joint in a pediatric patient with ASD patient. Preparations were performed to ensure favorable perioperative management by collecting information before admission from the patient’s parents and the teachers at his school for special needs education.

Children with ASD who undergo surgery require individualized supportive strategies and a multi-faceted approach to ensure optimal care (8). A lack of understanding and knowledge of ASD among healthcare providers is one of the factors interfering with patient access to appropriate services (9). Therefore, previous reports have emphasized the importance of collecting information about autistic characteristics from parents before hospitalization (5). However, no reports to date have emphasized the importance of collecting information from schools for special needs education.

Schools for special needs education serve children with comparatively severe disabilities. In these schools, children learn through a special curriculum while being surrounded by a sufficient number of teachers and various facilities and equipment that meet their needs (10). According to a Japanese survey of schools for special needs education conducted by Nishimura et al. (11), 45.3% of the children in these schools were medically diagnosed with ASD. Moreover, all of these schools had a curriculum for ASD, and the learning environment was devised for children with ASD. Therefore, ASD care at schools for special needs education may be established through education and individual trial and error. We have previously shown that interpersonal communication between children with ASD and the doctors and staff promotes positive emotions and reduces medical examination-related anxiety among these patients (12). For medical staff, who were not family members, the individualized communication methods employed by schoolteachers, who were also not family members, may be more specific, objective, and easier to apply to medical situations. In fact, we obtained a lot of concrete information from the school in this case (Table 1). Thus, information obtained from schools for special needs education may be useful for preparation of children with ASD.

Our report has a limitation. Because the patient’s school for special needs education is located nearby his welfare facility, there is a possibility that the information obtained from his school partially includes information from his welfare facility.

In conclusion, we present a case of successful perioperative management in a pediatric patient with ASD. We collected information available for preparation not only from family members but also from teachers at the patient’s school for special needs education. Thus, comprehensive information from the patient’s family and the educational environment may facilitate the process of preparing for perioperative management of pediatric patients with ASD.

## Data availability statement

The original contributions presented in the study are included in the article/supplementary material, further inquiries can be directed to the corresponding author.

## Ethics statement

The studies involving humans were approved by Ethical committee of Tottori University Hospital. The studies were conducted in accordance with the local legislation and institutional requirements. Written informed consent for participation in this study was provided by the participants’ legal guardians/next of kin. Written informed consent was obtained from the individual(s) for the publication of any potentially identifiable images or data included in this article.

## Author contributions

TO was responsible for the organization and coordination of the trial. YA was the chief investigator responsible for data analysis. YN and YM designed the trial. YA, TO, YN, and YM contributed to the writing of the final manuscript and met the ICMJE authorship criteria. All authors contributed to the article and approved the submitted version.
